# Müllerian clear cell carcinoma arising from sigmoid endometriosis with pericolic lymph node metastasis in the absence of adnexal disease: a case report

**DOI:** 10.3389/fonc.2026.1945284

**Published:** 2026-08-27

**Authors:** Matteo Girlando, Jose Manuel Garcia, Caterina Ravaioli, Antonio De Leo, Francesco Mezzapesa, Pierandrea De Iaco, Anna Myriam Perrone

**Affiliations:** 1 Division of Gynecologic Oncology, Scientific Institute for Research, Hospitalization and Care (IRCCS) Azienda Ospedaliero-Universitaria di Bologna Policlinico di Sant’Orsola, Bologna, Italy; 2 Department of Medical and Surgical Sciences (DIMEC), University of Bologna, Bologna, Bologna, Italy; 3 Solid Tumor Molecular Pathology Laboratory, Scientific Institute for Research, Hospitalization and Care (IRCCS) Azienda Ospedaliero-Universitaria di Bologna, Bologna, Italy

**Keywords:** clear cell carcinoma, endometriosis, extragenital endometriosis, Mullerian tumor, sigmoid colon

## Abstract

**Background:**

Malignant transformation of endometriosis is rare and most commonly involves the ovary. Extraovarian transformation, particularly in the gastrointestinal tract, is exceptional, and clear cell carcinoma (CCC) arising from intestinal endometriosis is rarely reported.

**Case presentation:**

We report a 64-year-old postmenopausal woman with no prior history of endometriosis in whom a sigmoid lesion was incidentally detected during follow-up for a presumed benign ovarian cyst. Colonoscopic biopsy revealed a poorly differentiated carcinoma with clear cell features, and immunohistochemistry (PAX8+, CK7+, HNF1β+, CK20−) supported a Müllerian origin. The patient underwent complete cytoreductive surgery (R0). Final histology confirmed Müllerian clear cell carcinoma arising from sigmoid endometriosis with pericolic lymph node metastasis (pT3N1M0). Integration of the immunophenotypic profile (PAX8+, CK7+, HNF1β+, CK20−), and demonstration of adjacent endometriosis supported the diagnosis of Müllerian clear cell carcinoma arising from endometriosis, fulfilling the Sampson and Scott criteria. She received adjuvant carboplatin and paclitaxel and is disease-free at 6 months.

**Conclusion:**

Müllerian CCC arising from intestinal endometriosis is extremely rare and may occur in postmenopausal women without known endometriosis. Accurate immunohistochemical diagnosis is essential to avoid misclassification and guide appropriate oncologic management.

## Introduction

Endometriosis is a chronic inflammatory condition affecting approximately 10% of women of reproductive age and is characterized by the presence of endometrial-like tissue outside the uterine cavity, leading to inflammation, fibrosis, and adhesions. Although traditionally considered a benign disease, endometriosis exhibits several features of malignancy, including invasive growth, neoangiogenesis, and immune evasion (1).

Malignant transformation of endometriosis is a rare event, occurring in approximately 0.5–2.5% of cases, and most commonly involves the ovary (2). Malignant transformation of endometriosis is thought to involve a multistep process driven by chronic inflammation and oxidative stress, together with the accumulation of molecular alterations affecting pathways involved in cell proliferation, survival, and apoptosis, including alterations in ARID1A and PI3K/AKT/mTOR signaling (3–6) Extraovarian transformation accounts for approximately 25% of cases and is usually associated with endometrioid histology, whereas clear cell carcinoma (CCC) represents a less frequent but clinically relevant subtype (7). The diagnosis of carcinoma arising from endometriosis is based on the Sampson and Scott criteria: 1) coexistence of benign endometriosis and carcinoma, 2) histologic continuity between them, and 3) exclusion of another primary tumor, along with histological evidence of a transition from benign endometriosis to carcinoma (2).

Müllerian CCC arising from intestinal endometriosis is exceptionally rare, particularly in the absence of ovarian involvement. Moreover, it may occur in postmenopausal women and in the absence of a known history of endometriosis, posing significant diagnostic challenges and a potential risk of misclassification as primary colorectal carcinoma. In these cases, differential diagnosis can be particularly challenging and requires integration of morphological and immunophenotypic findings with histopathological evidence of adjacent endometriosis.

In this context, we report a case of Müllerian clear cell carcinoma arising from sigmoid endometriosis with pericolic lymph node metastasis and no evidence of adnexal disease, providing detailed clinicopathological and immunohistochemical characterization and highlighting the diagnostic and therapeutic implications of this rare entity.

## Case presentation

**Figure 1:**
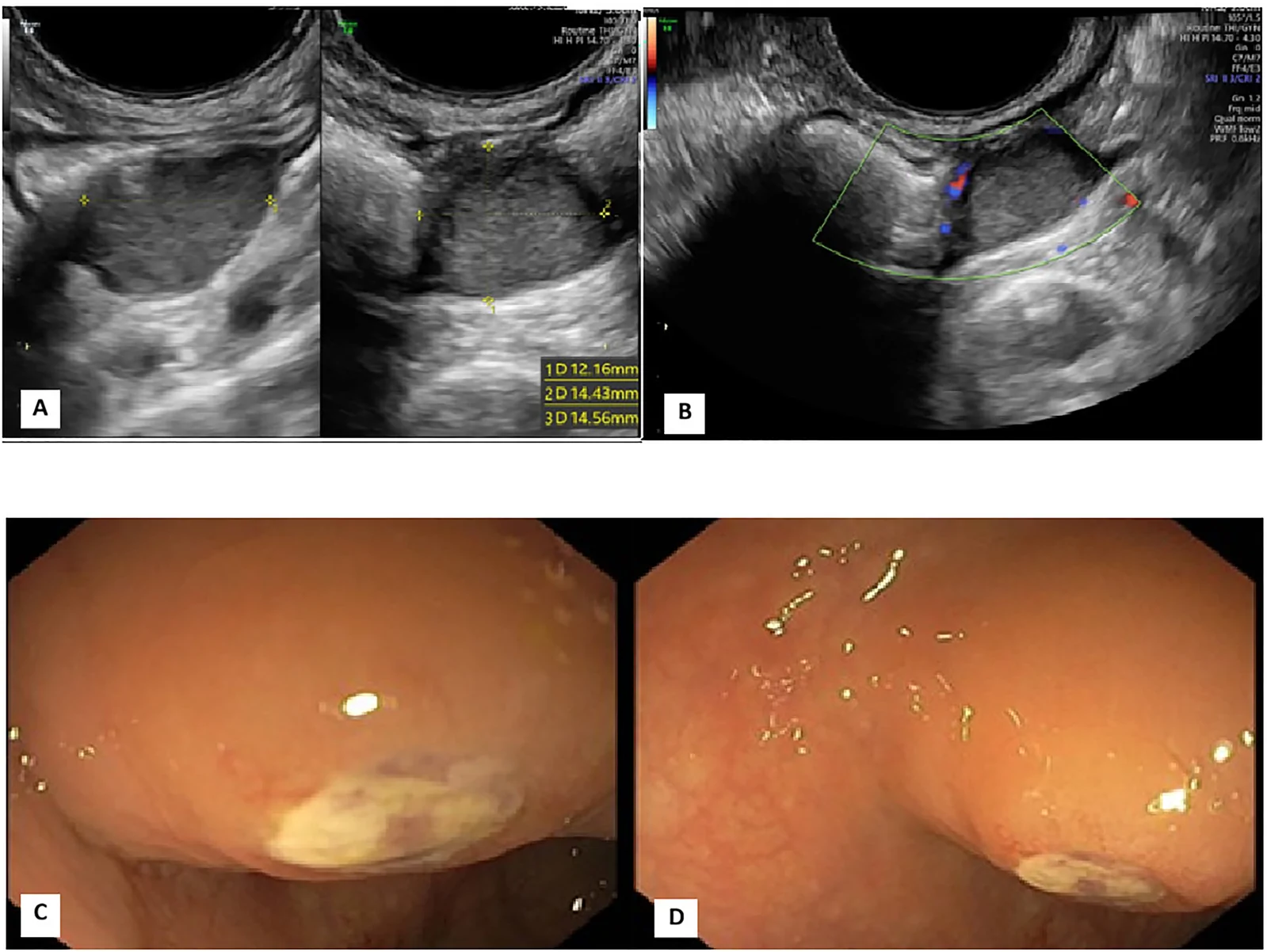
**(A, B)** Transvaginal ultrasound images of the lesion showing a solid mass arising from the lateral wall of the sigmoid colon, showing internal vascularization (CS2); **(C, D)** Endoscopic vision of an ulcerated sigmoid lesion.

A 64-year-old postmenopausal woman (BMI 23, two vaginal deliveries, spontaneous menopause at 53 years, no prior pelvic surgery, no history of hormone replacement therapy) with a history of hypertension and no prior diagnosis of endometriosis presented to the emergency department in May 2025 with hypertensive crisis and chest pain. Laboratory findings suggested acute myocardial injury; however, coronary angiography excluded significant coronary artery disease. The patient was discharged on antihypertensive therapy and scheduled for further investigations, including abdominal ultrasound. In June 2025, abdominal ultrasound revealed a suspicious left ovarian cyst, and the patient was referred to our tertiary referral center. Transvaginal ultrasound performed in July 2025 identified a unilocular solid cyst of the left ovary measuring 11 × 11 × 12 mm, with a 5 mm papillary projection and no vascularization (Color Score 1). The patient was asymptomatic, and a 4-month follow-up was planned. At follow-up, the ovarian lesion remained stable. However, a new 12 × 14 × 15 mm solid mass was identified arising from the lateral wall of the sigmoid colon, showing internal vascularization and synchronous movement with the adjacent bowel loop, suggesting an intestinal origin (**Figures 1A, B**). The patients was asymptomatic for abdominal pain or dyschezia. Contrast-enhanced computed tomography (CT) showed no evidence of ascites, lymphadenopathy, or peritoneal carcinomatosis and did not identify any rectal mass or other suspicious rectal lesion. Serum CA-125 was within the normal range (9 U/mL). Colonoscopy confirmed the presence of an ulcerated sigmoid lesion (**Figures 1C, D**), and biopsy revealed a poorly differentiated carcinoma with clear cell features and papillary architecture. Immunohistochemistry was positive for PAX8, CK7, and HNF1β, supporting a Müllerian origin. In January 2026, the patient underwent proctosigmoidectomy with total mesorectal excision, modified radical hysterectomy, bilateral salpingo-oophorectomy, omentectomy, peritoneal biopsies, and bilateral pelvic and para-aortic lymphadenectomy. Complete cytoreduction (R0) was achieved. The postoperative course was uneventful, and the patient was discharged on postoperative day 6 without complications. Final histopathological examination revealed Müllerian clear cell carcinoma diffusely infiltrating the sigmoid colon, with full-thickness involvement and lymphovascular invasion. One of seven pericolic lymph nodes was positive for metastatic disease (3.5 mm), while a total of 34 pelvic and para-aortic lymph nodes were negative. Surgical margins were free of disease. *Histopathological examination of the left ovarian lesion revealed a serous cystadenofibroma, with no evidence of malignancy.*

**Figure 2:**
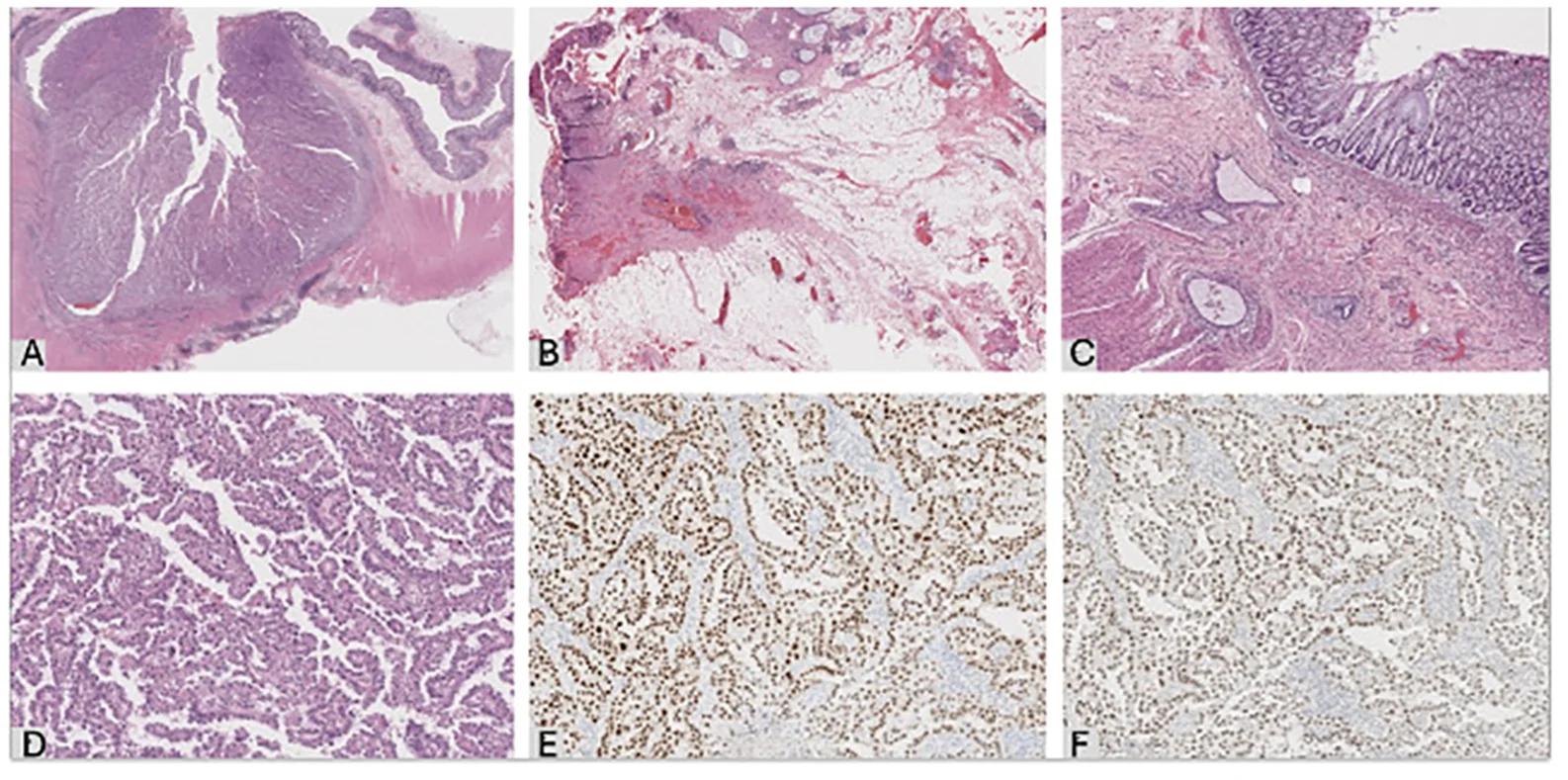
**(A)** Hematoxylin and eosin (H&E), low magnification (5×), showing clear cell carcinoma infiltrating the large bowel wall; **(B)** H&E, intermediate magnification (10×), showing endometriotic foci in the pelvic peritoneum; **(C)** H&E, high magnification (50×), showing endometriosis within the large bowel wall; **(D)** H&E, high magnification (100×), showing clear cell carcinoma; **(E)** Immunohistochemical staining (DAB, 100×) showing nuclear positivity for HNF1β; **(F)** Immunohistochemical staining (DAB, 100×) showing nuclear positivity for SOX17.

Immunohistochemical analysis (**Figure 2**) showed positivity for CK7 and SOX17 and negativity for CK20. ARID1A and PTEN expression were preserved, hormone receptors were negative, p53 showed a wild-type pattern, and mismatch repair proteins were proficient. The uterus and adnexa were free of malignancy. Foci of perivisceral endometriosis adjacent to the tumor were identified, fulfilling the Sampson and Scott criteria. The case was discussed at a multidisciplinary tumor board. In the absence of a dedicated staging system, the tumor was classified according to the colorectal TNM system as pT3N1M0 (stage IIIB). The patient completed adjuvant chemotherapy with carboplatin and paclitaxel and is currently alive and disease-free at 6 months of follow-up (**Table 1**).

**Table 1:** Clinical timeline according to CARE Check list.

Time	Clinical event
Initial presentation	Asymptomatic. Incidental finding of ovarian cysts after routine ultrasound
Diagnostic work-up	Ultrasound. CT scan. Colonoscopy.
Preoperative assessment	Multidisciplinary evaluation and treatment planning
Surgery	Total hysterectomy + Bilateral Salpingo-oophorectomy + sigmoid resection + peritonectomy + omentectomy + pelvic and lomboartic lymphadenectomy
Histopathological examination	Müllerian clear cell carcinoma diffusely infiltrating the sigmoid colon
Postoperative period	Postoperative course was uneventful, and the patient was discharged on postoperative day 6 without complications
Follow-up	The case was discussed at a multidisciplinary tumor board. the tumor was classified according to the colorectal TNM system as pT3N1M0 (stage IIIB). The patient was started on adjuvant chemotherapy with carboplatin and paclitaxel and is currently alive and disease-free at 4 months of follow-up

## Discussion

To our knowledge, this represents one of the very few reported cases of Müllerian clear cell carcinoma arising from sigmoid endometriosis with pericolic lymph node metastasis, and one of the most comprehensively characterized in terms of immunohistochemical profile.

A clinically relevant aspect of this case is the context in which the tumor developed. It demonstrates that malignant transformation of endometriosis may occur in the colon, in a postmenopausal patient, in the absence of a known history of endometriosis, and in the presence of completely normal ovaries. These findings challenge the traditional perception of endometriosis-associated malignancies as predominantly ovarian diseases affecting women of reproductive age. Instead, they support the concept that clinically silent or previously undiagnosed endometriotic foci may persist beyond menopause and retain the potential for malignant transformation. Consequently, Müllerian origin should be considered in the differential diagnosis of atypical colorectal tumors in women, regardless of age or prior clinical history.

Clear cell carcinoma involving the colon is an exceptionally rare entity and encompasses two biologically distinct subtypes: intestinal-type and Müllerian-type tumors (8). This distinction is clinically crucial, as these entities differ not only in histogenesis but also in diagnostic pathways and therapeutic management. Intestinal-type clear cell carcinoma is typically associated with conventional colorectal adenocarcinoma or adenomatous lesions and expresses markers of intestinal differentiation, such as CK20, CDX2, and CEA (8). In contrast, Müllerian-type tumors arise from ectopic endometrial tissue and display a characteristic immunophenotype, including CK7, PAX8, HNF1β, and SOX17 positivity (8), with absence of intestinal markers.

In the present case, the coexistence of endometriosis adjacent to the tumor, the Müllerian immunoprofile (CK7+/CK20−/PAX8+/SOX17+), and the absence of a primary gynecologic malignancy strongly support the diagnosis of Müllerian clear cell carcinoma arising from intestinal endometriosis, fulfilling Sampson and Scott criteria (9). This distinction is not merely academic but has direct clinical implications. Without appropriate immunohistochemical characterization, this tumor could have been misclassified as a primary colorectal carcinoma, potentially leading to an inappropriate therapeutic strategy.

The extreme rarity of this condition limits robust comparisons with the literature. Nevertheless, previously reported cases provide a useful framework for contextualizing the present findings (**Table 2**). Earlier reports by Finkelstein et al., Min et al., and Okazawa et al. described clear cell adenocarcinomas arising in rectal endometriosis, supporting the concept that ectopic endometrial tissue within the colorectal wall can undergo malignant transformation toward a clear cell phenotype (10–12). More recently, Gaia-Oltean et al. reported a Müllerian clear cell carcinoma developing within rectal endometriosis, further emphasizing the diagnostic importance of demonstrating an association between the tumor and adjacent endometriotic foci (13).

**Table 2:** Comparison between our case and the “twin” case by Lochner at al. (2025).

Features	Lochner et al. (2025) case	Our case
Location	Rectum	Sigmoid colon
Histological Type	Mullerian clear cell carcinoma	Mullerian clear cell carcinoma
Uterus & adnexa	Disease free	Disease free
Endometriosis	Yes, on the rectum wall	Yes, on the sigmoid wall perivisceral tissue and parietal peritoneum
Nodes metastasis	5 pericolic nodes	1perisigmoidnode
Lomboaorticnodes	Not specified	Negative
ARID1A	Mutationidentified	Preserved function
PTEN	Not specified	Preserved function
p53	Not specified	Wild-type
MMR	Not specified	Proficient
Surgery	Total hysterectomy + Bilateral Salpingo-oophorectomy+Proctosigmoidectomy	Total hysterectomy + Bilateral Salpingo-oophorectomy + sigmoid resection + peritonectomy + omentectomy + pelvic and lomboartic lymphadenectomy
Adjuvant chemotherapy	yes	yes
Outcome	Disease-free at 4 years follow-up	Disease-free at 4-month follow-up

Despite their common association with intestinal endometriosis, the reported cases show considerable heterogeneity in clinical presentation, extent of disease, surgical management, and pathological assessment (**Table 2**). Notably, most previously described tumors involved the rectum, whereas the present tumor arose in the sigmoid colon, highlighting that malignant transformation may occur at different sites along the rectosigmoid tract. Furthermore, lymph-node metastases were absent in several previously reported cases. The most comparable case is that reported by Lochner et al. (2025) (14) (**Table 2**), describing a Müllerian clear cell carcinoma arising from rectal endometriosis with pericolic lymph node metastases and no ovarian involvement. Our case confirms and expands these observations, demonstrating that lymphatic dissemination may occur even in tumors arising from apparently localized intestinal endometriotic foci. The presence of nodal involvement has important surgical implications. *Although the available evidence is limited to a very small number of reported cases and does not allow definitive recommendations regarding the optimal surgical approach, these findings suggest that oncologic resection including lymph node assessment should be considered when Müllerian clear cell carcinoma arising from intestinal endometriosis is suspected.* Limited or segmental resections without nodal assessment may be insufficient, given the potential for lymphatic spread. This reinforces the need to manage these tumors according to oncologic principles rather than as benign or borderline lesions.

Staging and management remain challenging due to the absence of dedicated guidelines. In clinical practice, staging is generally based on colorectal TNM classification (15), reflecting the anatomical origin and pattern of dissemination. However, treatment strategies are largely derived from gynecologic oncology, particularly ovarian clear cell carcinoma, given the shared biological features. This distinction is critical, as therapeutic approaches differ significantly depending on tumor origin. In this context, complete cytoreductive surgery followed by platinum-based chemotherapy represents the most widely adopted strategy (16).

From a therapeutic perspective, molecular overlap with ovarian clear cell carcinoma may open future avenues for targeted treatment strategies. Alterations involving chromatin remodeling genes such as ARID1A and signaling pathways such as PI3K/AKT/mTOR have been associated with potential sensitivity to targeted therapies and immune checkpoint inhibitors in Müllerian tumors (17, 18). Although these alterations were not identified in our case, the underlying biological similarity suggests that molecular profiling may play an increasingly important role in guiding treatment in selected patients.

Finally, this case highlights the importance of a multidisciplinary approach. The integration of imaging, endoscopy, pathology, and gynecologic oncology expertise was essential to achieve an accurate diagnosis and define the most appropriate management strategy. However it has also inherent limitations. Given the exceptional rarity of Müllerian clear cell carcinoma arising from intestinal endometriosis, the available evidence is largely restricted to individual case reports, limiting meaningful comparisons and precluding definitive conclusions regarding optimal surgical and systemic treatment. Furthermore, the relatively short follow-up period of 6 months in the present case does not allow assessment of long-term oncologic outcomes.

In conclusion, this case expands the current spectrum of endometriosis-associated extragonadal malignancies and underscores several clinically relevant points: the potential for malignant transformation in asymptomatic endometriosis, even in postmenopausal women; the critical importance of accurate diagnosis to avoid misclassification and inappropriate treatment; and the need for an oncologically adequate surgical and therapeutic approach tailored to Müllerian origin. Its rarity, diagnostic complexity, and therapeutic implications make this entity highly relevant and deserving of further investigation.

## Data Availability

The original contributions presented in the study are included in the article/Supplementary Material. Further inquiries can be directed to the corresponding authors.

## References

[1] Somigliana E Vigano' P Parazzini F Stoppelli S Giambattista E Vercellini P . Association between endometriosis and cancer: a comprehensive review and a critical analysis of clinical and epidemiological evidence. Gynecol Oncol. (2006) 101:331–41. doi: 10.1016/j.ygyno.2005.11.033 16473398

[2] Mezzapesa F Dondi G Coada CA De Leo A de Terlizzi F Strigari L . Two possible entities of endometriosis-associated ovarian cancer: correlated or incidental? Int J Gynecol Cancer Off J Int Gynecol Cancer Soc. (2025) 35:101634. doi: 10.1016/j.ijgc.2025.101634 39955191

[3] Giannini A Massimello F Caretto M Cosimi G Mannella P Luisi S . Factors in Malignant transformation of ovarian endometriosis: a narrative review. Gynecol Endocrinol. (2024) 40:2409911. doi: 10.1080/09513590.2024.2409911 39445672

[4] Yamamoto S Tsuda H Takano M Tamai S Matsubara O . PIK3CA mutations and loss of ARID1A protein expression are early events in the development of cystic ovarian clear cell adenocarcinoma. Virchows Arch. (2012) 460:77–87. doi: 10.1007/s00428-011-1169-8 22120431

[5] Hablase R Kyrou I Randeva H Karteris E Chatterjee J . The “road” to Malignant transformation from endometriosis to endometriosis-associated ovarian cancers (EAOCs): An mTOR-centred review. Cancers (Basel). (2024) 16:2160. doi: 10.3390/cancers16112160 38893278 PMC11172073

[6] Kajiyama H Suzuki S Yoshihara M Tamauchi S Yoshikawa N Niimi K . Endometriosis and cancer. Free Radic Biol Med. (2019) 133:186–92. doi: 10.1016/j.freeradbiomed.2018.12.015 30562557

[7] Gadducci A Lanfredini N Tana R . Novel insights on the Malignant transformation of endometriosis into ovarian carcinoma. Gynecol Endocrinol Off J Int Soc Gynecol Endocrinol. (2014) 30:612–7. doi: 10.3109/09513590.2014.926325 24905724

[8] Remo A Grillo F Mastracci L Fassan M Sina S Zanella C . Clear cell colorectal carcinoma: Time to clarify diagnosis. Pathol Res Pract. (2017) 213:447–52. doi: 10.1016/j.prp.2017.02.013 28285963

[9] Sampson JA . Endometrial carcinoma of the ovary, arising in endometrial tissue in that organ. Arch Surg. (1925) 10:1–72. doi: 10.1001/archsurg.1925.01120100007001 33631893

[10] Alexander Finkelstein MD Gillian Helen Levy MD Pei Hui MP Department of Pathology, Yale University School of Medicine, New Haven, CT . . doi: 10.1309/LMJCTYEH9S22VQXW

[11] Min KW Koh YW Ryu YJ Hong SM Kim KR Sung CO . Primary clear cell adenocarcinoma arising from rectal endometriosis. In: Dig. Endosc. Seoul: Kyueng-Whan Min from the University of Ulsan College of Medicine. (2013). doi: 10.1111/den.12008 23362987

[12] Okazawa Y Takahashi R Mizukoshi K Takehara K Ishiyama S Sugimoto K . A case of clear cell adenocarcinoma arising from endometriosis of the rectum treated by laparoscopic surgery. Int J Surg Case Rep. (2014) 5:979–83. doi: 10.1016/j.ijscr.2014.10.034 25460452 PMC4276269

[13] Gaia-Oltean AI Boitor-Borza D Simedrea VC Braicu V Pop LA Micu R . Rectal clear cell carcinoma arising from endometriosis: Case report and literature review. Diagn (Basel). (2025) 15:1936. doi: 10.3390/diagnostics15151936 40804901 PMC12346751

[14] Lochner R Nakayama J Steinhagen E Bagby C Cui M . Müllerian clear cell carcinoma arising from rectal endometriosis with pericolonic lymph node metastasis. Virchows Arch. (2024) 486:1073–7. doi: 10.1007/s00428-024-03864-y 38977467

[15] American Joint Committee on Cancer (AJCC) . AJCC Cancer Staging Manual, 8th Edition. Available online at: https://www.facs.org/quality-programs/cancer-programs/american-joint-committee-on-cancer/cancer-staging-systems/ajcc-cancer-staging-system-products/ (Accessed April 20, 2026).

[16] National Comprehensive Cancer Network . Ovarian Cancer/Fallopian Tube Cancer/Primary Peritoneal Cancer. (Version 4.2026). Available online at: https://www.nccn.org/professionals/physician_gls/pdf/ovarian.pdf (Accessed April 20, 2026). 10.6004/jnccn.2024.0052 39413835

[17] De P Dey N . Mutation-driven signals of ARID1A and PI3K pathways in ovarian carcinomas: Alteration is an opportunity. Int J Mol Sci. (2019) 20:5732. doi: 10.3390/ijms20225732 31731647 PMC6888220

[18] Shen J Ju Z Zhao W Wang L Peng Y Ge Z . ARID1A deficiency promotes mutability and potentiates therapeutic antitumor immunity unleashed by immune checkpoint blockade. Nat Med. (2018) 24:556–62. doi: 10.1038/s41591-018-0012-z 29736026 PMC6076433

